# The Burden and Trend of Blood-Borne Pathogens among Asymptomatic Adult Population in Akwatia: A Retrospective Study at the St. Dominic Hospital, Ghana

**DOI:** 10.1155/2017/3452513

**Published:** 2017-10-18

**Authors:** Sylvester Yao Lokpo, Mavis Popuelle Dakorah, Gameli Kwame Norgbe, James Osei-Yeboah, Godwin Adzakpah, Isaac Sarsah, John Gameli Deku, Innocent Afeke, Emmanuel Akomanin Asiamah, Nana Yaw Barimah Manaphraim, Isaac Asare, Bright Justice Ayidzoe, Emmanuel Alote Allotey, Emmanuel Agbeko Nani, Paul Amoah

**Affiliations:** ^1^Department of Medical Laboratory Sciences, School of Allied Health Sciences, University of Health and Allied Sciences, Ho, Ghana; ^2^Laboratory Department, St. Dominic Hospital, Akwatia, Eastern Region, Ghana; ^3^School of Allied Health Sciences, University of Health and Allied Sciences, Ho, Ghana; ^4^Department of Health Information Management, University of Cape Coast, Cape Coast, Central Region, Ghana; ^5^Laboratory Department, Ada East District Hospital, Ghana Health Service, Ada, Greater Accra Region, Ghana; ^6^Clinical Biochemistry Unit, Laboratory Department, Volta Regional Hospital, Ghana Health Service, Ho, Volta Region, Ghana

## Abstract

**Background:**

This study was aimed at evaluating the seroprevalence and trend of blood-borne pathogens (HIV, HCV, HBV, and Syphilis) among asymptomatic adults at Akwatia during a four-year period (2013–2016).

**Materials and Methods:**

The study was a retrospective analysis of secondary data of blood donors who visited the hospital from January 2013 to December 2016. Archival data from 11,436 prospective donors was extracted. Data included age, sex, and place of residence as well as results of infectious markers (HIV, HBV, HCV, and Syphilis).

**Results:**

The prevalence of blood-borne pathogens in the donor population was 4.06%, 7.23%, 5.81%, and 10.42% for HIV, HBV, HCV, and Syphilis infections, respectively. A significant decline in HBV and HCV infections was observed in the general donor population and across genders. HIV infection rate remained steady while Syphilis infections recorded a significantly increasing trend, peaking in the year 2015 (14.20%). Age stratification in HBV infection was significant, peaking among age group 40–49 years (8.82%).

**Conclusion:**

Asymptomatic blood-borne pathogen burden was high among the adult population in Akwatia. Gender variations in HBV, HCV, and Syphilis infections in the cumulative four-year burden were observed. Awareness needs to be created, especially in the older generation.

## 1. Introduction

Blood-borne pathogens are a threat to human lives and of public health importance in the developing world [1, 2]. Sub-Sahara Africa is projected to have the highest burden of blood-borne infections among the adult population [3, 4]. Blood-borne pathogens are infectious agents present in blood and these principally may include Hepatitis B Virus (HBV), Hepatitis C Virus (HCV), Human Immunodeficiency Virus (HIV), and Syphilis [1]. Complications such as liver fibrosis, cirrhosis, end-stage liver disease, hepatocellular carcinoma (HCC), and mortality due to liver pathology as well as lowered immune system that allows life-threatening opportunistic infections could arise from prolonged and untreated blood-borne infections [5]. Previous studies have found it challenging to estimate the exact burden of these infections due to high cost of participants recruitment, inability to select a representative sample, poor laboratory procedures, and the asymptomatic and often latent nature of the disease prior to clinical presentation [6]. Pregnant women and people with tattooed bodies were used in certain studies to represent the population which does not give a true reflection of the infectious burden in the general population [7, 8]. To estimate the prevalence and trend of blood-borne infections in the general adult asymptomatic population in Akwatia, the current study used retrospective data of prospective blood donors who visited the St. Dominic Hospital, during a four-year period from 2013 to 2016 as a proxy population.

## 2. Methods

### 2.1. Study Design and Study Site

The study was a retrospective analysis of secondary data of prospective blood donors who visited the St. Dominic Hospital's Blood Bank, Akwatia, from January 2013 to December 2016. The study included male and female participants aged between 17 and 56 years whose complete records were available for review. Demographic parameters included age, sex, and place of residence. Results of infectious markers (HIV, HBV, HCV, and Syphilis) screening using rapid diagnostic test kits from a total of 11,436 blood donors were obtained from the archives at the blood bank.

### 2.2. Data Analysis

Data collected were entered into Microsoft Excel 2013 spreadsheet and validated for entry errors. Data was presented as frequency and proportions. Differences between proportions and trend analysis were done using Fisher's exact test and chi square test for trends where appropriate. At all times, a *p* value < 0.05 was considered as statistically significant. IBM Statistical Package for the Social Sciences (SPSS Inc., Chicago, USA) (http://www.spss.com) version 22.00 was used for data analysis.

### 2.3. Ethical Consideration

Written approval for the study was sought and obtained from the management of St. Dominic Hospital, Akwatia. Analysis of the data was anonymous and nonlinked, and no donor names were retrieved from the archives.

## 3. Results

For the four (4) years under review, a total of 11,436 prospective blood donors at the St. Dominic Hospital, Akwatia, were included in the study. Among these donors, 1192 (10.41%) tested positive for Syphilis, 827 (7.23%) for HBV, 664 (5.81%) for HCV, and 464 (4.06%) for HIV. The review observed a significantly year-on-year decreasing trend of viral hepatitis infection (HBV, HCV). As seen in Table 1, HBV infection recorded a reduction from 9.85% in 2013 to 4.46% in 2016 and HCV reduced from 8.15% in 2013 to 3.28% at the end of the year 2016. The reported HIV infection remained steady throughout the four-year period under review (*p *for trends = 0.5426). In general, Syphilis infection rate recorded an increasing trend, peaking in the year 2015 at 14.20% (see Table 1).

Among the six hundred and eighty-five (685) female blood donors for the four (4) years under review, 33 representing 4.82% tested positive for HBV, 56 (8.18%) for HCV, 31 (4.53%) for HIV, and 55 for Syphilis (8.03%). Significantly, a decreasing year-on-year trend in HCV infection was observed among the female donor population (*p *for trends = 0.0014). Though not statistically significant, percentage year-on-year reduction in HIV infection was recorded. A statistically stable rate was observed for HBV and Syphilis infections, with their seropositivity peaking at 6.09% and 11.68%, respectively, in 2015 (see Table 2).

Among the ten thousand seven hundred and forty (10,740) male blood donors for the four (4) years under review, the viral infections recorded were 794 (7.39%), 608 (5.66%), and 433 (4.03%) for HBV, HCV, and HIV, respectively, and the bacterium infection (VDRL) was 1137 (10.59%). Significant reduction in the year-on-year rate was observed for hepatitis infection (*p *for trends < 0.0001). The HIV infection rate was statistically stable. An observed increasing trend was recorded for Syphilis infection peaking in 2015 at 14.35% (see Table 3).

**Figure 1:**
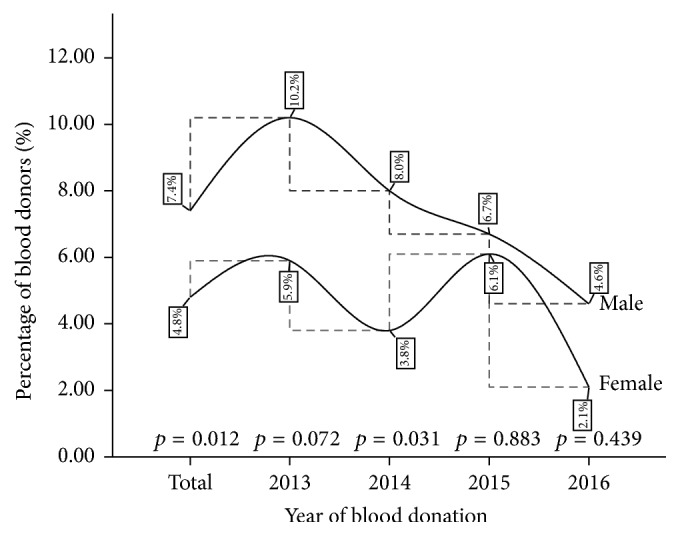
A four-year gender variation in HBV infection among blood donors at the St. Dominic Hospital Akwatia* (p is significant at 0.05).*

Significant gender variation in HBV infection was observed for the cumulative four-year infection rate (*p* = 0.012), with infection preponderance tilted toward the male blood donor population (Figure 1). Significant higher infection burden among the males was also recorded in the year 2014 (*p* = 0.031). Though the male donor group recorded a higher percentage HBV infection (4.6%) in 2016 compared to their female counterparts (2.1%), the difference was statistically not significant (see Figure 1).

**Figure 2:**
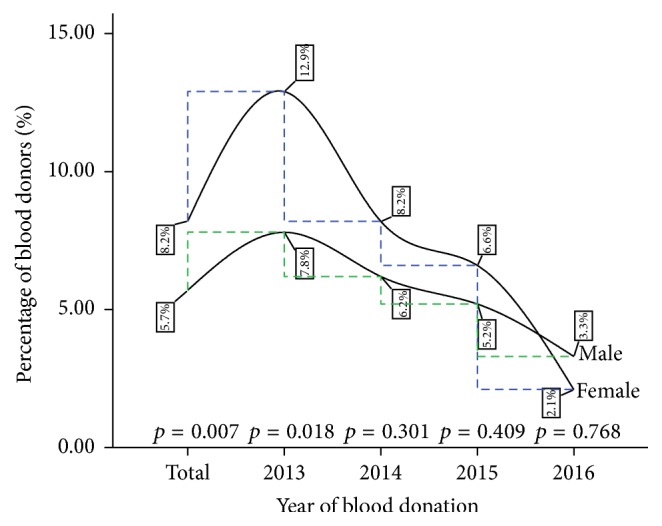
A four-year gender variation in HCV infection among blood donors at the St. Dominic Hospital Akwatia* (p is significant at 0.05). *

The cumulative percentage of HCV burden was found to be significantly higher among the female blood donors (7.4%) compared to their male counterparts (4.8%) (*p* = 0.012). In the initial year of the review (2013), significant difference in HCV infection burden existed, tilted toward the female donor population; this gap became narrower year after year, ending with a lower percentage burden among the females in 2016 (see Figure 2).

**Figure 3:**
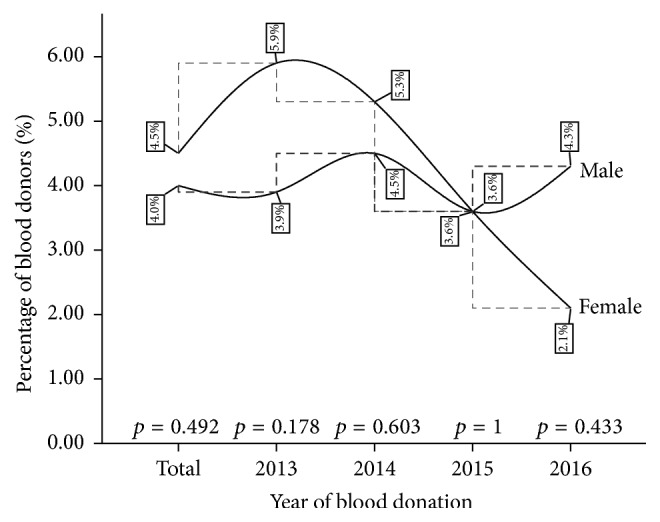
A four-year gender variation in HIV infection among blood donors at the St. Dominic Hospital Akwatia* (p is significant at 0.05).*

Although no significant gender variation in HIV infection rate was observed for both the cumulative infection burden (*p* = 0.492) and the year-on-year comparison throughout the review period, the pattern of HIV infection rate was greater among the females in the first two years (2013 and 2014) of the period under review and the HIV infection rate was the same among both males and females in 2015 but lower among the females in 2016 (see Figure 3).

**Figure 4:**
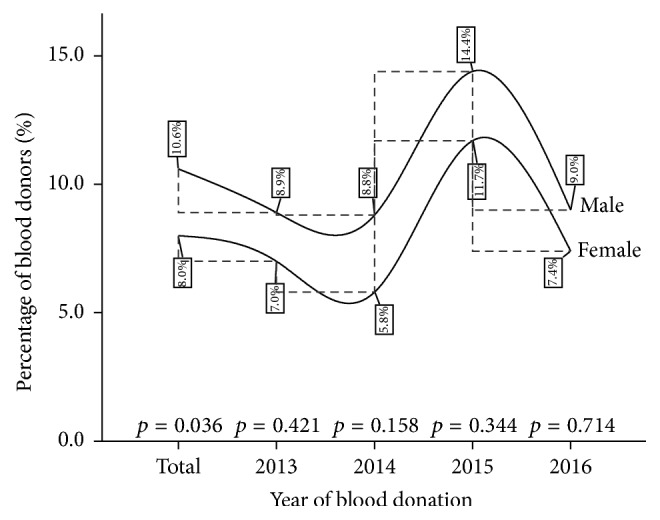
A four-year gender variation in Syphilis infection among blood donors at the St. Dominic Hospital Akwatia* (p is significant at 0.05).*

As seen from Figure 4, a higher preponderance for Syphilis infection was recorded among the male blood donor population in the cumulative four-year infection burden (*p* = 0.036). However, the year-on-year comparisons showed no significant gender variation in Syphilis infection (see Figure 4).

Majority of the donor population were within the 20–39-year-old bracket (80.34%). Significant age stratification in HBV infection was observed, with HBV infection rate ranging from 6.68% among blood donors within the 20–29-year age bracket and peaking among those within the 40–49-year age bracket (8.82%) (*p* = 0.0102). Syphilis infection was lowest among the younger donor population (6.27%) peaking in the 40–49-year age bracket (16.18%) (*p* < 0.0001). No significant age distribution difference in infection burden was recorded for HCV and HIV (see Table 4).

In the four years under review, 2862 (25.03%) of all potential blood donors at the St. Dominic Hospital were deferred due to reactions to serological markers. Among this deferred group, 16.14% were deferred due to expression of viral markers. Multiple infection of blood-borne pathogens was low among the blood donor population. No coinfection among the four serological markers added up to 1% of the infectious burden among the donor population presenting with infection (see Table 5).

## 4. Discussion

In the current study, the four years under review (2013–2016) recorded a total of 11,436 units of blood that were screened at the St. Dominic Hospital in Akwatia. The prevalence of blood-borne pathogens among this asymptomatic adult population was 4.60%, 7.23%, 5.81%, and 10.41% for HIV, HBV, HCV, and Syphilis infections, respectively. Previous results of high prevalence of these infectious pathogens have been reported in Ghana. Ampofo et al. [9] reported a prevalence of 3.8%, 8.4%, and 13.5% for HIV, HCV, and Syphilis, respectively. Nkrumah et al. [10] recorded 13.8% HBV and 9.4% HCV. Walana et al. [11] reported 9.6% HBV, 4.4% HCV, and 4.9% HIV and Faustina et al. [12] recorded 8.5% prevalence of Syphilis. In other African countries, high burden of blood-borne infections has been reported in Burkina Faso [13], South Ethiopia [1], and Northwestern Nigeria [14]. Lower seroprevalence rates of blood-borne infections have been reported in other jurisdictions. Yang et al. [15] in China reported 0.08%, 0.51%, 0.20%, and 0.57% prevalence for HIV, HBV, HCV, and Syphilis, respectively. Farshadpour et al. [16] in Iran reported a prevalence of 0.15% HBV, 0.1% HCV, and 0.004% HIV. Huda and Nasir [17] recorded 1.42% and 0.1% for HBV and HCV infection rates in a Bangladeshi population.

Several factors are attributed to account for the difference in the burden of blood-borne pathogens across different populations. These include, but are not limited to, socioeconomic status, geographical location, sanitary conditions, education, number of sexual partners, sensitivity of test kits used, cultural practices such as female genital mutilation, immunization status, tattooing, bloodletting exercises, and selection criteria [10, 18–20].

The prevalence of the infectious markers among the female population was 4.53%, 4.82%, 8.18%, and 8.03% whereas, in the male donor population, the rate was 4.03%, 7.39%, 5.66%, and 10.59% for HIV, HBV, HCV, and Syphilis infections, respectively. The current findings suggest that the male subpopulation in our study were more likely to record a higher HBV and Syphilis infections compared to their female counterparts with the exception of HIV and HCV infection rates which recorded a female preponderance. The reason for this inconsistent infection pattern observed in the present study is not clear but could partly be attributed to the inequality in the recruitment of participants with respect to gender. The males in this study outnumber their female counterparts with a ratio of 1 : 15; hence the estimated burden of blood-borne pathogens in the male population could be skewed. However, our results are partly in agreement with the report by Bisetegen et al. [1] where males recorded higher prevalence of HBV and HCV compared to the higher burden of HIV and Syphilis infections observed among their adult female peers.

In the general population, a significant year-on-year decreasing trend of viral hepatitis infection (HBV and HCV) was observed (Table 1). HBV infection rate recorded a reduction from 9.85% in 2013 to 4.46% in 2016 and HCV infection reduced from 8.15% in 2013 to 3.28% at the end of 2016. In the female population, a significant decreasing year-on-year trend in HCV infections (*p* = 0.0014) was observed with a statistically stable rate for HBV infection peaking at 6.09% in 2015 (Table 2). In the male population, the reduction in the year-on-year infection rate was observed for both HBV and HCV (*p* < 0.0001) (Table 3). Additionally, when the variations in the HBV and HCV prevalence were compared across gender, there was a significant difference in the cumulative four-year burden and in the year 2013 (Figures 1 and 2).

The general decline in the year-on-year trend analysis of viral hepatitis (HBV and HCV) in this study is consistent with previous reports of studies among asymptomatic adult Ghanaians. Nkrumah et al. [10] reported a fall in the prevalence of HBV and HCV from 13.8% to 6.9% and 11.1% to 7.0% between the years 2006 and 2008, respectively, at Agogo, in the Ashanti Region. Walana et al. [11] observed a drop from 11.0% to 8.2% and 4.5% to 2.3%, respectively, for HBV and HCV prevalence during a 3-year study period (2010–2012) at Kintampo, in the Brong Ahafo Region. No specific reason can be given as an attributable factor from the current data. Explaining the possible reasons for the observed phenomenon, Nkrumah et al. [10] posited that the decreasing rate of HBV and HCV infections could result from horizontal transmission which is often associated with low endemicity rather than vertical transmission. In their opinion, horizontal transmission is linked to age, socioeconomic conditions, socioprofessional status, and risky behaviours such as scratching the back of carriers, biting fingernails, and sharing bath towels among others. Farshadpour et al. [16] suggested that declining trends could reflect a fall in the rates of these infections in the general population.

There has been a concerted global effort to drastically reduce the burden of HIV since the advent of the pandemic decades ago. Some strategies included the flagship Millennium Development Goal Six (6) which aimed to combat infectious diseases including HIV/AIDS by 2015. Although many countries have experienced decreases in HIV/AIDS mortality and infections, other countries have had slowdowns in the rate of change in infections [21]. In this study, the burden of HIV remained steady throughout the four years (*p* = 0.5426). This steady trend was also observed in the male and female subpopulations (Tables 1, 2, and 3) as well as when gender comparison was made (Figure 3). The stable trend of HIV prevalence observed during the study period could reflect the trend in the general Ghanaian population. In Ghana, the estimated national HIV prevalence rates from Sentinel surveys have been fairly stable ranging from 2.0% in 2010 to 2.4% in 2016 [22]. This could suggest that the strategies put in place by the Government of Ghana through its implementing agency, National AIDS Control Programme (NACP) to reduce HIV burden, though initially seeing a significant fall, have not recorded any significant decrease within the last few years.

In the current study, the four-year cumulative prevalence and year-on-year prevalence recorded an increasing trend of Syphilis infection peaking in 2015 at 14.20% (Table 1). Similar increasing trend was observed in the male subpopulations, while a stagnation in the trend was revealed in the female population peaking in the year 2015 at 11.68% (Table 2). The upsurge in the trend of Syphilis infection concurs with those reported earlier by Salawu et al. [4], Yang et al. [15], and Fasola et al. [23]. In contrast, Tessema et al. [24] and Sachdeva et al. [25] recorded a downward trend of Syphilis infection in various populations. It is not apparent what could be driving the rise in Syphilis prevalence in the current study. However, Sachdeva et al. [25] postulated that the rising tendency could be a window of an evolving risk behaviour and changing lifestyle among the general populace.

Age is considered an independent risk factor for acquiring blood-borne infections with varied results of associated specific age category [26, 27]. Some studies have reported infections in individuals under 30 years of age while others reported vulnerability to the infectious markers in persons over 30 years of age in various populations [12, 26, 28, 29]. In the current study, a significant age stratification in HBV infection was observed, with rates ranging from 6.68% among participants within 20–29-year age bracket, which peaked among those within the 40–49-year age bracket (8.82%) (*p* = 0.0102). Syphilis infection was lowest among the younger donor population (6.27%) but peaked in the 40–49-year age bracket at 16.18% (*p* < 0.0001) (Table 4).

It is believed that blood-borne infectious agents share common modes of transmission and risk groups [24]. This could lead to simultaneous occurrence of HBV, HCV, HIV, and Syphilis infections presenting in the individual either a dual or multiple infection depending on which combination is acquired. Multiple infections may include HBV-HCV, HBV-HIV, HIV-HCV, HBV-Syphilis, and HIV-Syphilis [10, 13]. In the four years under review, the prevalence of multiple infection was low among participants. No coinfection among the four serological markers sum up to 1% of the infectious burden (Table 5). Results of low rate of coinfections of blood-borne pathogens similar to this study have been reported previously in other studies [10, 11, 14]. In our study, the coinfections observed were HBV-HCV [68 (0.59%)], HBV-Syphilis [80 (0.70%)], and HCV-Syphilis [79 (0.69%)]. Evidence exist that individuals with coinfections, particularly HBV and HCV infections, could develop fulminant hepatitis with severe histological lesions leading to poor response to therapy [4]. The presence of HCV could suppress HBV replication leading to low HBV-DNA levels, decreased HBV-DNA polymerase activity, concealing Hepatitis B surface antigen expression in blood thus presenting as occult hepatitis [4].

## 5. Conclusion

Asymptomatic blood-borne pathogen burden was high among the adult population in Akwatia. Gender variations were recorded in HBV, HCV, and Syphilis infections. Older generation was most vulnerable to blood-borne infections. There is the need to create awareness to reduce the burden especially in the older generation.

## Figures and Tables

**Table 1:** Year-on-year trends in infectious markers among blood donors at the St. Dominic Hospital Akwatia, Ghana.

Year	Total	2013	2014	2015	2016	*p* value
Blood donors	11,436	2,529	3,111	3,507	2,289	
HBV	827 (7.23)	249 (9.85)	241 (7.75)	235 (6.70)	102 (4.46)	<0.0001
HCV	664 (5.81)	206 (8.15)	198 (6.36)	185 (5.28)	75 (3.28)	<0.0001
HIV	464 (4.06)	103 (4.07)	141 (4.53)	125 (3.56)	95 (4.15)	0.5426
Syphilis	1192 (10.42)	222 (8.78)	268 (8.61)	498 (14.20)	204 (8.91)	0.0021

Data is presented as frequency and corresponding percentage in parenthesis. HBV: Hepatitis B Virus; HCV: Hepatitis C Virus; HIV: Human Immunodeficiency Virus. *p* for trends is significant at 0.05.

**Table 2:** Year-on-year trends in infectious markers among female blood donors at the St. Dominic Hospital Akwatia, Ghana.

Year	Total	2013	2014	2015	2016	*p* value
Blood donors	685	186	208	197	94	
HBV	33 (4.82)	11 (5.91)	8 (3.85)	12 (6.09)	2 (2.13)	0.4189
HCV	56 (8.18)	24 (12.90)	17 (8.17)	13 (6.60)	2 (2.13)	0.0014
HIV	31 (4.53)	11 (5.91)	11 (5.29)	7 (3.55)	2 (2.13)	0.1021
Syphilis	55 (8.03)	13 (6.99)	12 (5.77)	23 (11.68)	7 (7.45)	0.2653

**Table 3:** Year-on-year trends in infectious markers among male blood donors at the St. Dominic Hospital Akwatia, Ghana.

Year	Total	2013	2014	2015	2016	*p* value
Blood donors	10,740	2,343	2,903	3,310	2,184	
HBV	794 (7.39)	238 (10.16)	233 (8.03)	223 (6.74)	100 (4.58)	<0.0001
HCV	608 (5.66)	182 (7.77)	181 (6.23)	172 (5.20)	73 (3.34)	<0.0001
HIV	433 (4.03)	92 (3.93)	130 (4.48)	118 (3.56)	93 (4.26)	0.8848
Syphilis	1137 (10.59)	209 (8.92)	256 (8.82)	475 (14.35)	197 (9.02)	0.0043

Data is presented as frequency and corresponding percentage in parenthesis. HBV: Hepatitis B Virus; HCV: Hepatitis C Virus; HIV: Human Immunodeficiency Virus. *p* for trends is significant at 0.05.

**Table 4:** Infectious markers among blood donors at the St. Dominic Hospital Akwatia, Ghana, stratified by age.

Parameter	Donors	HBV	HCV	HIV	Syphilis
<20 years	909 (7.97)	64 (7.04)	59 (6.49)	47 (5.17)	57 (6.27)
Twenties	6508 (57.07)	435 (6.68)	392 (6.02)	249 (3.83)	573 (8.80)
Thirties	2654 (23.27)	213 (8.03)	125 (4.71)	104 (3.92)	345 (13.00)
Forties	1168 (10.24)	103 (8.82)	79 (6.76)	54 (4.62)	189 (16.18)
≥50 years	165 (1.45)	12 (7.27)	8 (4.85)	9 (5.45)	24 (14.55)
*p* value	*Nd*	0.0102	0.3854	0.7228	<0.0001

Data is presented as frequency and corresponding percentage in parenthesis. HBV: Hepatitis B Virus; HCV: Hepatitis C Virus; HIV: Human Immunodeficiency Virus. *p* is significant at 0.05. nd = *p* value estimation not done.

**Table 5:** Patterns of multiple infection of blood-borne serological markers among blood donors at the St. Dominic Hospital, Akwatia.

Parameter	Infectious marker		Viral infection
At least one	2862 (25.03)		1846 (16.14)
Single	2592 (22.67)		1738 (15.20)
Two	256 (2.24)		107 (0.94)
Three	13 (0.11)		1 (0.01)
Four	1 (0.01)		—

Patterns of coinfection of blood-borne serological markers			

Infectious marker	HCV	HIV	Syphilis

HBV	68 (0.59)	15 (0.13)	80 (0.70)
HCV		27 (0.24)	79 (0.69)
HIV			32 (0.28)

Data is presented as frequency and corresponding percentage in parenthesis. HBV: Hepatitis B Virus; HCV: Hepatitis C Virus; HIV: Human Immunodeficiency Virus.
